# An optimized intermolecular force field for hydrogen-bonded organic molecular crystals using atomic multipole electrostatics

**DOI:** 10.1107/S2052520616007708

**Published:** 2016-07-16

**Authors:** Edward O. Pyzer-Knapp, Hugh P. G. Thompson, Graeme M. Day

**Affiliations:** a Department of Chemistry, University of Cambridge, Lensfield Road, Cambridge CB2 1EW, England; b School of Chemistry, University of Southampton, Highfield, Southampton SO17 1BJ, England

**Keywords:** lattice energy, crystal structure prediction, multipoles, polarization, electrostatics

## Abstract

We present a re-parameterization of a popular intermolecular force field for describing intermolecular interactions in the organic solid state. Specifically we optimize the performance of the exp-6 force field when used in conjunction with atomic multipole electrostatics. We also parameterize force fields that are optimized for use with multipoles derived from polarized molecular electron densities, to account for induction effects in molecular crystals. Parameterization is performed against a set of 186 experimentally determined, low-temperature crystal structures and 53 measured sublimation enthalpies of hydrogen-bonding organic molecules. The resulting force fields are tested on a validation set of 129 crystal structures and show improved reproduction of the structures and lattice energies of a range of organic molecular crystals compared with the original force field with atomic partial charge electrostatics. Unit-cell dimensions of the validation set are typically reproduced to within 3% with the re-parameterized force fields. Lattice energies, which were all included during parameterization, are systematically underestimated when compared with measured sublimation enthalpies, with mean absolute errors of between 7.4 and 9.0%.

## Abstract 2

An empirically parameterized intermolecular force field is developed for crystal structure modelling and prediction. The model is optimized for use with an atomic multipole description of electrostatic interactions.

## 1. Introduction   

The role of computational modelling in understanding the molecular organic solid state is developing rapidly, and computer simulations are key to understanding a wide range of properties of molecular solids, such as lattice energies (Nyman & Day, 2015 ▸), mechanical properties (Karki *et al.*, 2009 ▸), solubility (Palmer *et al.*, 2008 ▸, 2012 ▸), lattice dynamics (Li *et al.*, 2010 ▸; King *et al.*, 2011 ▸) and molecular dynamics (Gavezzotti, 2013 ▸), disorder (Habgood *et al.*, 2011 ▸), conformational preferences (Thompson & Day, 2014 ▸) and polymorphism (Cruz-Cabeza & Bernstein, 2014 ▸). The field of crystal engineering is concerned with relationships between molecular structure and crystal structure, whose computational embodiment is the ever-developing field of crystal structure prediction (Day *et al.*, 2009 ▸; Bardwell *et al.*, 2011 ▸; Day, 2011 ▸; Price, 2014 ▸).

In the past few years, the accessibility of high-performance computing has increased the use of periodic electronic structure calculations to study molecular crystals. However, most modelling of the molecular solid state continues to rely on force field methods, in which interatomic interactions are described by analytic functions whose parameters are derived either from *ab initio* calculations or empirical fitting to reproduce experimentally determined properties. A wide variety of such force fields are available and some of the most successful intermolecular force fields for modelling the organic solid state were developed by D. E. Williams. The latest of these was the W99 intermolecular force field (Williams, 1999 ▸, 2001*a* ▸,*b* ▸), which was developed by fitting the parameters of a Buckingham (exp-6) repulsion–dispersion model to reproduce the observed structures and measured sublimation enthalpies of sets of organic molecular crystal structures.

During parameterization of W99, Williams modelled electrostatic interactions between molecules using an atomic partial charge model supplemented by off-nuclear partial charges placed to describe anisotropic features of the electron density surrounding atoms in molecules. Here, we present a revision to Williams’ W99 parameters, fitted to perform optimally when combined with a distributed multipole representation of the molecular charge distribution. Such atomic multipole models yield a more faithful description of directional intermolecular interactions than atomic partial charges by correctly describing the long-range electrostatic potential arising from anisotropic features of the charge density, such as π-electron density and lone pairs. The limitations of describing a molecular charge distribution by atomic partial charges are most apparent when modelling hydrogen bonding, whose strength and directionality is inadequately described by such simple models (Buckingham & Fowler, 1985 ▸; Coombes *et al.*, 1996 ▸; Day *et al.*, 2005 ▸). Atomic multipoles are gaining popularity in force field modelling now that molecular modelling software capable of handling the required anisotropic atom–atom interactions and the resulting non-central forces is available (Price *et al.*, 2010 ▸; Rasmussen *et al.*, 2007 ▸). Their use has become particularly common in the field of organic molecular crystal structure prediction, where the relative energies of alternative crystal packings must often be resolved to about 1 kJ mol^−1^ or less (Day *et al.*, 2004 ▸, 2009 ▸; Price & Price, 2006 ▸; Mohamed *et al.*, 2008 ▸; Bardwell *et al.*, 2011 ▸; Vasileiadis *et al.*, 2012 ▸; Nyman & Day, 2015 ▸).

Despite being parameterized using an atomic partial charge electrostatic model, the W99 force field has been coupled with atomic multipole electrostatics with good success in the prediction of organic crystal structures (Kazantsev *et al.*, 2011 ▸; Baias *et al.*, 2013 ▸; Pyzer-Knapp *et al.*, 2014 ▸) and crystal properties (Day *et al.*, 2001 ▸, 2003 ▸, 2006 ▸). However, our experience is that the description of some hydrogen-bond interactions is unbalanced in a W99 + multipoles model, leading to unphysical geometries and unreliable energies for some types of hydrogen bonding. This is due, in part, to the way that empirical parameterization of W99 has absorbed the effects of many contributions to intermolecular energies into its parameters, charge transfer and charge penetration being particularly important in strong hydrogen bonds. The more realistic atomic multipole electrostatics have different demands of the exp-6 parameters than the more simplistic atomic charge model.

Therefore, we have focused our re-parameterization on the description of intermolecular hydrogen bonds in organic molecular crystals. Another weakness of W99 for hydrogen-bonding molecules that we seek to address is that the original fitting was performed separately to oxohydrocarbons and azahydrocarbons; none of the molecules used in the original parameterization contained either N—H⋯O or O—H⋯N hydrogen bonds. It is for crystals containing these hydrogen bonds that we have experienced the most problems in our applications of W99.

The re-parameterization is performed by fitting to a set of 186 experimentally determined low-temperature crystal structures and 53 measured sublimation enthalpies of molecules containing as diverse a set of *D*—H⋯*A* (*D*, *A* = O, N) hydrogen bonds as possible. Low-temperature crystal structures were chosen for parameterization, so that the resulting force field parameters are affected as little as possible by thermal expansion. Thus, the force field can be used with simulation methods that include the effects of temperature explicitly.

We also develop versions of the atom–atom potential in which the hydrogen-bonding parameters are optimized for use with atomic multipoles derived using a polarizable continuum model (PCM; Cossi *et al.*, 1998 ▸) of molecular polarization in crystals. The use of a dielectric continuum to mimic the environment of a molecule in a crystal has been proposed as an efficient method of including polarization effects into lattice energy calculations (Cooper *et al.*, 2008 ▸). However, the parameters of an empirically fitted exp-6 repulsion–dispersion model derived with unpolarized electrostatics already absorb some average polarization in crystal structures, resulting in double-counting if used with an explicit model of polarization. Therefore, the parameters of the exp-6 model are refitted to be consistent with the use of the PCM model of polarization.

## 2. Methods   

Our aim in empirically determining the best set of parameters to describe intermolecular interactions remains the same as that stated by Williams: ‘Our optimum intermolecular force field is one which gives the best fits to observed crystal structures and heats of sublimation. The goodness-of-fit is determined by minimization of the crystal energies using the force field to be tested, and comparing the resulting relaxed structures with the observed ones’ (Williams, 1999 ▸). Here, we describe the form of the force field, our strategy in optimizing the adjustable parameters and the selection of structures and energies to which we have parameterized.

### 2.1. Functional form of the force field   

We evaluate the total intermolecular contribution to a crystal’s lattice energy as the sum over atom–atom interactions
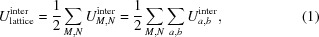
where 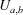 represents the interaction between atoms *a* and *b* belonging to molecules *M* and *N*, respectively. The form of the atom–atom interaction is largely the same as that described by Williams (1999 ▸, 2001*a* ▸,*b* ▸)
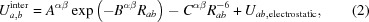
where *a* and *b* are atoms of type α and β, respectively. The first two terms describe a spherical-atom model that, while often referred to as the repulsion–dispersion model, must effectively describe all non-electrostatic contributions to the intermolecular interaction. The values of the parameters *A*, *B* and *C* depend on the atom types of the interacting atoms and are the parameters which are empirically fitted to structural and energetic data.

To limit the number of independent parameters in atom–atom force fields, it is common to use combining rules to relate the repulsion–dispersion parameters for heteroatomic interactions to the parameters describing homoatomic interactions. The following combining rules were used by Williams in the W99 force field
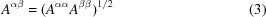


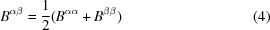


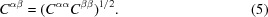



### 2.2. Electrostatic models   

The charge distribution on a molecule is described by a set of multipole moments, 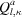, on each atomic site, *a*, where κ refers to one of the 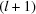 components of an atomic multipole moment of rank *l*. The intermolecular electrostatic energy is given by a sum over multipole–multipole interactions
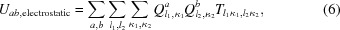
where the interaction functions, 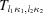, capture the radial (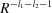) and angular dependence of the multipole–multipole interaction, as well as incorporating the factor of 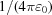. These interaction functions are tabulated elsewhere (Stone, 2013 ▸).

These multipole–multipole interactions are now implemented in various software packages, including *DMACRYS* (Price *et al.*, 2010 ▸), *TINKER* (Ren & Ponder, 2003 ▸), *ORIENT* (Stone *et al.*, 2002 ▸) and *AMBER* (Case *et al.*, 2005 ▸).

In this work, atomic multipoles are derived from the calculated molecular charge density with the original distributed multipole analysis (DMA) method (Stone, 1981 ▸), using the *GDMA* software (Stone, 1999 ▸). Molecular calculations have been performed using *GAUSSIAN*09 (Frisch *et al.*, 2009 ▸) at the B3LYP/6-31G** and B3LYP/6-311G** levels of theory. Atomic multipoles up to *l* = 4 (hexadecapole) are included on all atoms. The cost of calculations using the level of electrostatics here is approximately 8–10 times the cost of using a simpler atomic point charge model (Sagui *et al.*, 2004 ▸; Day *et al.*, 2005 ▸).

We also calculated atomic multipoles from single molecule calculations performed using the same functional and basis sets, with the molecule embedded within a PCM model of the polarizing environment of the crystal. We took a value for the dielectric constant of all molecular crystals to be 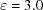 in these PCM calculations.

Molecular geometries were kept at the geometry found in the experimentally determined crystal structures, apart from *X*—H bond lengths in structures from X-ray diffraction, which were standardized to mean bond lengths seen in neutron diffraction crystal structures (Allen *et al.*, 1987 ▸). Hydrogen positions in crystal structures determined from neutron diffraction were left as-is.

All lattice energy calculations were performed with the *DMACRYS* software (Price *et al.*, 2010 ▸), using a quasi-Newton–Raphson minimization of unit-cell parameters and rigid molecule coordinates (orientations and center of mass positions). Ewald summation is used for charge–charge, charge–dipole and dipole–dipole interactions, while all higher electrostatic terms up to 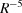, as well as non-electrostatic terms, are summed to a 30 Å direct space cutoff on separation between molecular centers of mass.

A final detail of the W99 force field relates to *X*—H bond ‘foreshortening’: as suggested by Williams, the interaction center for all H atoms is shifted 0.1 Å towards the atom to which it is covalently bonded (Williams, 2001*a* ▸). This centers the interaction at approximately the maximum in charge density, rather than the nuclear position of H atoms (Starr & Williams, 1977 ▸). We maintain this foreshortening throughout this work: the exp-6 site and multipole expansion site for H atoms are shifted to the foreshortened position.

### 2.3. Basis set effects   

The choice of basis set is known to have a strong influence on calculated molecular electrostatic moments (Halkier *et al.*, 1999 ▸; Hickey & Rowley, 2014 ▸). Much of the previous parameterization of force fields, and crystal structure modelling of molecular crystals has relied on electrostatic models derived from relatively small, polarized, double-zeta Gaussian basis sets. Double-zeta basis sets tend to underestimate molecular dipole moments and it has been shown that errors in calculated molecular dipoles are decreased significantly by using a triple-zeta basis set (Hickey & Rowley, 2014 ▸). The optimized empirical parameters of repulsion–dispersion models parameterized with electrostatics derived from a small basis set must absorb some of the effects of the errors in electrostatics.

We can, therefore, expect the optimized set of exp-6 parameters to differ with the basis set used to derive the electrostatic model. For this reason, we have considered the influence of basis set on the parameterization itself. Separate exp-6 parameter sets are derived for use with the B3LYP/6-31G**, B3LYP/6-311G** electrostatic models and their corresponding PCM models: B3LYP/6-31G** (PCM, 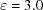) and B3LYP/6-311G** (PCM, 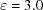). We refer to the revised W99 parameter sets as W99rev631, W99rev6311, W99rev631P and W99rev6311P, respectively.

### 2.4. Structure selection   

Experimentally determined crystal structures and measured sublimation enthalpies were compiled for fitting and testing of the force field. Parameterization and validation sets of crystal structure data were selected from searches of the Cambridge Structural Database (CSD; Allen, 2002 ▸). CSD refcodes are used to refer to structures throughout this work.

The W99 force field includes parameters for carbon, nitrogen, oxygen and hydrogen, and force field typing depends on atomic number as well as the atom’s bonding environment. We maintain the same atom typing as used in the original definition of W99 (Williams, 2001*a* ▸). Our focus in this work was the re-parameterization of hydrogen-bonding interactions which, in terms of the W99 atom typings, are interactions involving one of three polar H-atom types that can act as hydrogen-bond donors:

1. H2 – hydrogen in an alcoholic group;
2. H3 – hydrogen in a carboxylic group;
3. H4 – hydrogen in an N—H group;

and six types of possible hydrogen-bond acceptors

1. N1 – triple bonded nitrogen;
2. N2 – nitrogen with no bonded hydrogen (excluding triple bonded N);
3. N3 – nitrogen with one bonded hydrogen;
4. N4 – nitrogen with two or more bonded H atoms;
5. O1 – oxygen bonded to one other atom;
6. O2 – oxygen bonded to two other atoms.

The *ConQuest* (Bruno *et al.*, 2002 ▸) software was used to search the CSD for organic molecular crystal structures containing each combination of hydrogen-bond donor and acceptor. For the purposes of searching for structures, we defined a hydrogen bond as being present using fairly loose geometrical parameters, allowing any *D*—H⋯*A* angle in the range from 100 to 180° and allowing an interatomic separation between the non-H atoms, *D* and *A*, up to the sum of van der Waals radii + 0.2 Å.

Structures were restricted to molecules containing C, H, N and O, excluding polymeric structures, structures displaying any form of disorder and high-pressure crystal structures. The disorder of proton positions within dimers of carboxylic acid groups was ignored during energy minimizations (*i.e.* the reported H-atom position was used). Hydrate crystal structures were excluded. Because of the importance of H-atom positions in hydrogen bonds, crystal structures determined from neutron diffraction were preferred over X-ray diffraction, where available. We included only structures with crystallographic *R*-factors of less than 0.07; we originally aimed for an *R*-factor limit of 0.05, but this was increased to provide a better coverage of hydrogen-bond types.

So that the force-field parameters describe the temperature-free lattice energy surface as closely as possible, initial searches were performed for low-temperature crystal structures determined below 100 K; the *T* < 100 K restriction had to be relaxed to find sufficient structures with each type of hydrogen bond, but the influence of higher-temperature structures during parameter fitting was decreased, *see below*.

The training set contained 186 crystal structures. Sufficient crystal structures (at least 5 for each hydrogen-bond type) were found with 15 of the 18 hydrogen-bond acceptor–donor combinations. Insufficient crystal structures with the combinations H2⋯N4, H3⋯N3 and H3⋯N4 were found, so we are not able to re-parameterize these hydrogen bonds. The infrequency of these combinations in observed crystal structures makes their omission in this re-parameterization unimportant; where required, parameters from the original W99 can be used.

We initially sought training set crystal structures which each contained only one type of hydrogen bond, so that each hydrogen-bond parameter could be parameterized independently. This was only possible for eight hydrogen-bond types. For the remaining seven hydrogen-bond types, sufficient crystal structures for parameterization could only be found by including structures with multiple types of hydrogen bond. Therefore, we chose an order to perform the parameterization so that only one hydrogen bond had to be parameterized at a time (Table 1 ▸). The eight hydrogen-bond types in round 1 were parameterized using crystal structures containing only that type. The resulting parameter values were fixed during round 2 when a further four hydrogen-bond types were fitted, and similarly for rounds 3 and 4.

Measured sublimation enthalpies were found for 53 of the parameterization crystal structures and this data was included in the force field optimization. This data is listed in the supplementary information.

We also compiled a validation set of 129 low-temperature crystal structures using the same selection criteria as the parameterization set, which included examples of all but one of the hydrogen-bond donor–acceptor combinations (Table 1 ▸). The exception is the H3⋯N1 hydrogen bond (carboxylic acid to nitrile nitrogen), which is found in so few crystal structures that all structures of suitable quality had to be used during parameterization.

Diagrams and CSD reference codes of all molecules in the training set are provided as supporting information.

### 2.5. Fitting the potential   

#### 2.5.1. Definition of the target function   

To fit the hydrogen-bonding parameters of the force field, we adjust the exp-6 parameters to minimize a target function comprising terms describing the structural distortion of crystal structures upon lattice-energy minimization and how well measured heats of sublimation are reproduced by the calculated lattice energies. Structural data provide the force field with information regarding the position of the local minimum on the lattice energy surface, which is a balance of all interatomic forces in the crystal structure. A successful force field should result in a local minimum in the lattice energy very close to the structure of an experimentally determined crystal structure. Including sublimation enthalpies in the fitting function ensures that the atom–atom parameters give a realistic overall depth of the energy minimum.

As a measure of the structural change upon lattice energy minimization, we use a structural discrepancy factor based on that defined by Filippini & Gavezzotti (1993 ▸)
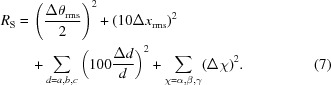


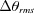
 is the root mean squared rigid-body rotation (in °) of molecules in the unit cell and 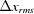 is the rigid body displacement (in Å) of molecular centers of mass during lattice energy minimization. *a*, *b*, *c* and α, β and γ are the unit-cell lengths and angles, respectively. The factors of 2, 10 and 100 are included to give roughly equal magnitude to each of the terms during a typical lattice energy minimization. We average the molecular rotations and displacements over all molecules in the unit cell so that the magnitude of 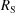 does not grow systematically with increasing numbers of independent molecules in a crystal structure. For some solvate crystal structures containing nearly linear solvent molecules (acetonitrile and methanol), the rotational term corresponding to these molecules was excluded from the computation of 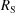, since very large 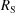 values could be obtained by only moving H atoms whose positions are of low accuracy in the experimentally determined crystal structures.

By ignoring changes in molecular conformation and intramolecular energy between gas and crystal phases, using equipartition values for molecular rotational and translational contributions to the ideal gas phase enthalpy, and equipartition internal energy contributions from rigid molecule phonon vibrations in the crystal phase, we can approximately relate the lattice energy and enthalpy of sublimation of a crystal as
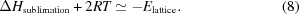
Therefore, for crystal structures with measured sublimation enthalpies, we can estimate the error in the calculated lattice energy as
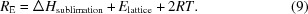
This differs from Williams’ parameterization, who omitted the 2*RT* temperature correction. Since the 2*RT* correction has been shown to be an acceptable estimate of the true thermal correction (Otero-de-la-Roza & Johnson, 2012 ▸), we include 2*RT* here for correctness and in the hope of improving the fit to measured energies.

When using the polarized charge densities (calculated within a PCM model of the crystal environment), the calculated lattice energy is corrected for the relaxation energy of the molecular charge density between PCM and the gas phase (the difference in electronic energy of the polarized and unpolarized molecules).

The overall target function, *R*, that we seek to minimize combines the energetic (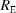) and structural (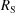) terms described above
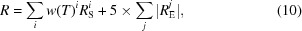
where *i* runs over all crystal structures in a given training set and *j* runs over all crystal structures with associated sublimation data in the training set. The weighting factor for 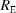 takes into account the expected errors in structure and energy. We set our target for energetic discrepancies as 4 kJ mol^−1^ and target for 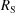 as 55 (which corresponds approximately to typical differences between lattice-energy-minimized and room-temperature crystal structures; Filippini & Gavezzotti, 1993 ▸). The weighting of 5 (with 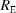 measured in kJ mol^−1^) makes typical errors in 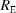 equal to one of the terms in 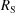.


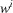
 is a temperature-dependent weighting of the structural discrepancy, giving most importance to low-temperature crystal structures during the parameterization
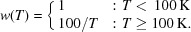
This weighting reduces the influence of thermal expansion on the force-field parameters, so that the force field describes as closely as possible the temperature-free potential energy surface.

Finally, due to the importance of H-atom positions in hydrogen bonds, we doubled the weight of all structures determined by neutron diffraction relative to structures determined by X-ray diffraction, to increase the contribution of structures with accurate H-atom positions during parameterization.

#### 2.5.2. Fitting of the parameters   

Due to the high correlation between 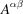 and 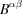, it is generally not possible to empirically parameterize both parameters of the exponential repulsion simultaneously. Therefore, we kept 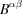 fixed at Williams’ values and only re-parameterized 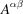 for all hydrogen-bond interactions (where α or β = H2, H3 or H4).

The dispersion coefficients, 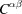, for any interactions involving polar H atoms (H2, H3 and H4) are set to zero in Williams’ parameterization of W99 (Williams, 2001*a* ▸,*b* ▸); the electron density associated with these atoms is so small and has a low polarizability that they contribute very little to intermolecular dispersion interactions. We made a similar observation to Williams: allowing non-zero 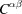 for interactions involving polar H atoms leads to a negligible improvement in reproducing the crystal structures and sublimation enthalpies in our parameterization set, at the cost of doubling the number of parameters requiring optimization. We therefore kept 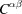 as zero for all hydrogen bonding H⋯*X* interactions that we have re-parameterized here. This leaves only the repulsive pre-exponential, 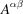, to parameterize for each hydrogen-bond interaction.

The optimum 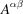 parameters were found by performing line searches of the exp-6 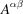 parameters, lattice-energy minimizing all crystal structures in the parameterization set at each 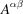 value. The value of the fitting function, *R*, was obtained by comparison of the resulting lattice energy minima to experimental structures and sublimation enthalpies. Initial parameterization of 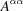 (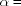 H2, H3 or H4) was investigated using the combining rules for H⋯*X* interactions. Finding that significant improvement could be obtained by abandoning the combining rules, parameterization was performed separately for all 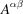 (α = H2, H3 or H4, β = O1, O2, N1, N2, N3, N4). The minimum for each parameter was located to within 1 eV (0.5% to 3% of their final, optimized values).

## 3. Results   

### 3.1. Combining rules *versus* explicitly parameterized cross-terms   

We initially attempted parameterization of the hydrogen-bond repulsion parameters, 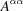 (α = H2, H3 or H4), using the combining rules [equations (3)–(5) [Disp-formula fd4] [Disp-formula fd5]] to relate heteroatomic interaction parameters to the parameters describing homo­atomic interactions.

However, we observed that the best performing value for the H-atom repulsion parameter, 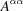, varies significantly with the nature of the *acceptor* atom. Most noticeably, crystal structures with O and N atoms as hydrogen-bond acceptors are best reproduced using quite different parameters for the hydrogen repulsion. For example, in the case of H4 as the donor atom, nitrogen hydrogen-bond acceptor atoms tended to require a higher value for the repulsion than oxygen acceptors. Although less pronounced, we also observed differences between atom types of the same element: hydrogen bonding with N1 and N2 acceptors are better modelled with a higher H4 repulsion than N3 and N4 acceptors, while O1 acceptors on average want a lower repulsion than O2.

These findings are at odds with previous force-field parameterization experience (Coombes *et al.*, 1996 ▸), but make physical sense when we consider that the exponential repulsion parameters absorbs the effects of all short-range interactions, including charge transfer in hydrogen bonds, whose contribution should not be expected to behave as an average of charge transfer in homoatomic interactions. Given these observations, we made the decision to explicitly parameterize each of the donor–acceptor pairs without use of combining rules, *i.e.* the repulsion parameter is fitted independently for each hydrogen-bond combination.

### 3.2. Final parameters   

Final parameters resulting from training of the force field using all four multipolar electrostatic models are listed and compared with those in the original W99 force field in Table 2 ▸. These should be used with the original W99 parameters (as listed in the supporting information) for all other interactions.

The optimized parameters differ significantly from the original W99 parameters, particularly in some of the heteroatomic hydrogen bonding (O—H⋯N and N—H⋯O) interactions. There are also noticeable differences between parameters optimized using 6-31G** and 6-311G** basis sets: the 6-311G** electrostatics generally require a larger repulsion between hydrogen-bond donor and acceptor atoms, to balance the stronger electrostatic interactions resulting from the more accurate electron density.

The inclusion of polarization in the electrostatic model also results in enhanced electrostatic interactions, which leads to larger repulsion parameters to model the hydrogen bonds. Thus, the repulsion parameters are up to a factor of 3 larger in the W99rev6311P force field, compared with W99rev631.

### 3.3. Validation   

To evaluate the performance of the optimized parameter sets outside of the training set of crystal structures, a validation set of crystal structures was selected from the CSD. The validation set covers the range of hydrogen-bond types quite well (Table 1 ▸). Since crystal structures containing only one type of hydrogen bond were preferred when selecting structures for the parameterization set, the validation set mainly contains structures with multiple types of hydrogen bonds.

The performance of the force fields is evaluated by how well the validation structures are reproduced upon lattice-energy minimization. As a general measure of structural changes during lattice energy minimization, we examine the structural drift value, 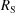, as defined in equation (7), for the validation crystal structures. We also examine the changes in crystal density, unit-cell parameters and the geometries (lengths and angles) of hydrogen bonds.

Far fewer measured sublimation enthalpies are available than crystal structures. Therefore, nearly all reliable sublimation enthalpies were used in parameterization. In the absence of a separate validation set of energies, we examine the performance of the force fields against all crystal structures with measured sublimation enthalpies (53 from the parameterization set + 5 additional structures not used during parameterization).

For comparison with the newly parameterized models, calculations were performed on the validation set and all crystal structures with sublimation enthalpies using the original W99 force field, coupled with atomic multipoles derived from a B3LYP/6-31G** charge density.

### 3.4. Reproduction of experimental structures   

Firstly, we note that with the original W99 potential, 22 of the validation crystal structures failed to find a lattice energy minimum due to a particularly poor description of the relevant hydrogen-bond interaction. The failed optimizations are not spread evenly amongst the different hydrogen-bond types; all failures contain the H3⋯N2 interaction, which is typically a hydrogen bond between carboxylic acid and a pyridine ring. Upon inspection, we find that these failed optimizations result from an unphysical shortening of the hydrogen-bond interaction. With each of the re-parameterized potentials, all validation set crystal structures successfully reached an energy minimum. In the following, the failed optimizations are omitted from analysis of the original W99, but included for all of the other force fields.

#### 3.4.1. Overall structural drift   

The first measure on which the new potentials are assessed is the overall structural drift, 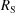. Since the starting point for each lattice energy minimization was a well defined experimental structure, a smaller structural drift indicates a better performance for the potential.

**Figure 1:**
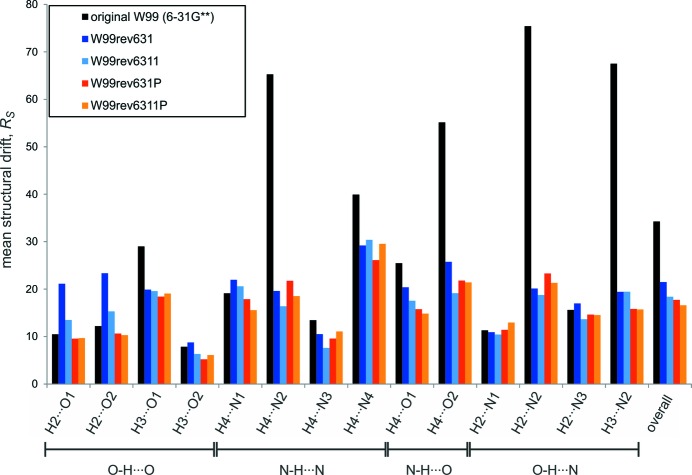
Mean structural drift, 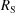, during lattice energy minimization of the validation crystal structures using each force field, and broken down by hydrogen-bonding type.

The mean values of 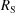 across the validation structures (Fig. 1 ▸) demonstrate that, on average, crystal structures of hydrogen-bonded organic molecules are reproduced more accurately by the re-paramaterized force fields. Mean values of 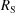 are reduced by over a third, from 34.2 with the original W99 to 21.5 with W99rev631, which uses the same electrostatic model. Mean 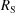 values decrease further with the larger 6-311G** basis set electrostatics (W99rev6311, mean 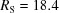) and again with the two models that include polarization of the molecular electrostatics (W99rev631P, mean 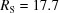, and W99rev6311P, mean 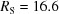), for which the mean 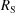 is approximately half that with the original W99.

To help interpret these improvements, assuming that 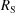 has equal contributions from each term [see equation (7)], the best mean 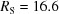 (using W99rev6311P) corresponds to changes in lattice parameters of approximately 0.7%, unit-cell angles changes of 0.7°, molecular rotations of 4° and center of mass displacements of 0.2 Å.

The improvement in reproducing observed crystal structures is most pronounced for certain hydrogen-bond types: H4⋯N2; H4⋯O2; H2⋯N2 and H3⋯N2, where the performance of the original W99 with atomic multipole electrostatics was poor. The errors are more consistent across hydrogen-bond types with the re-parameterized force fields.

#### 3.4.2. Unit-cell parameters and densities   

**Figure 2:**
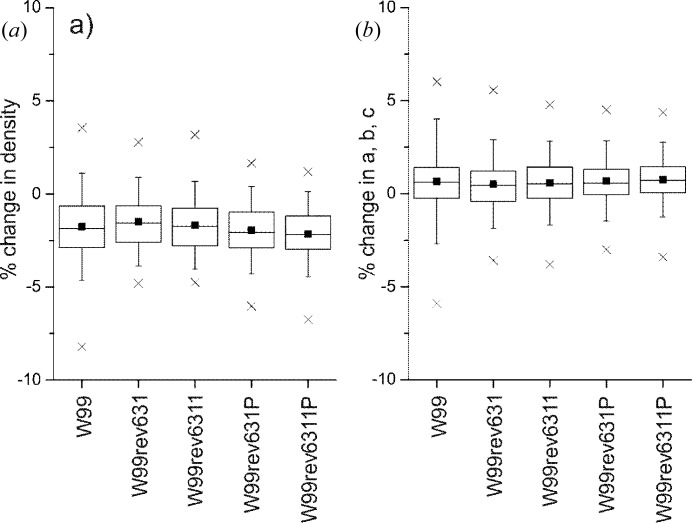
Box plots showing the changes in (*a*) density and (*b*) lattice parameters (*a*, *b* and *c*) during lattice-energy minimization of the validation set of crystal structures with the force fields. Horizontal lines of the box show the first, second (median) and third quartiles. Filled squares show the mean and whiskers indicate one standard deviation above and below the mean. Crosses indicate the maximum deviations. Structures that failed to find a minimum with the original W99 are excluded from the W99 statistics only.

Crystal densities of the lattice-energy minimized crystal structures are, on average, about 2% lower than the densities of the experimentally determined crystal structures. The box plots in Fig. 2 ▸(*a*) show that the slight expansion of crystal structures is consistent across the validation set, with standard deviations of the density change of 1.9% with W99, decreasing to between 1.5 and 1.6% with the re-parameterized models. The decrease in density may be due to the original W99 parameterization against ambient temperature structures, so that all non-hydrogen-bonding interactions have absorbed some thermal expansion into the parameters, which expands the low-temperature structures in the validation set.

Similarly, individual lattice parameters are reproduced well. Mean errors in lattice parameters are between 0.53 and 0.76% for the five force fields (Fig. 2 ▸ *b*). The mean error is not improved in the re-parameterized force fields, but the spread of errors decreases, demonstrating that re-parameterization has led to more consistently performing force fields.

#### 3.4.3. Hydrogen-bond geometries   

Finally, since we have focused our improvement on parameters that describe hydrogen-bonding interactions, we examine how well the force fields reproduce the geometries of hydrogen bonds in the validation set of crystal structures.

**Figure 3:**
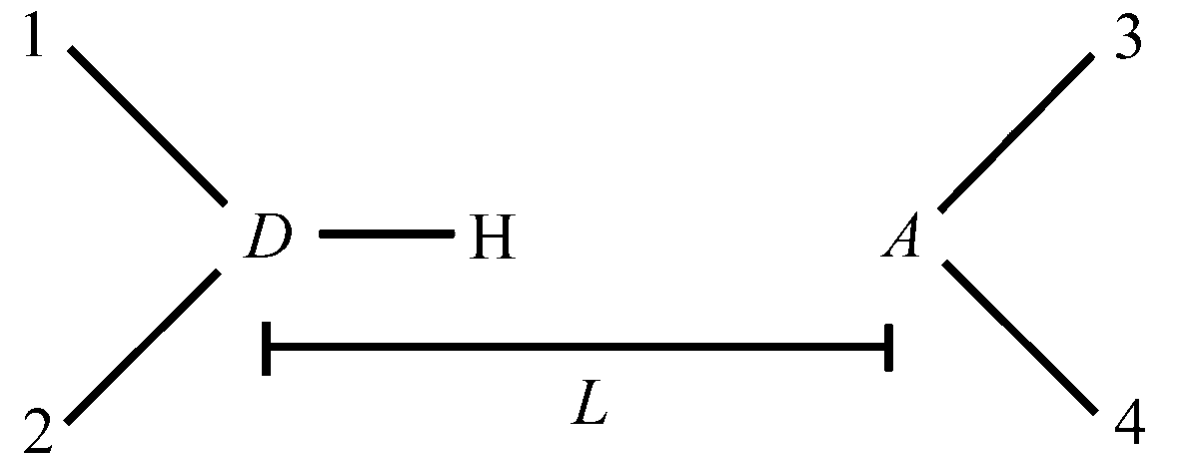
Definition of the hydrogen-bond length, *L*, used. Up to four hydrogen-bond angles are considered: ∠ 1—*D*—*A*; ∠ 2—*D*—*A*; ∠ 3—*A*—*D* and ∠ 4—*A*—*D*.

Given that most of the structures in the validation set have been determined from X-ray diffraction, the accuracy of positions of H atoms can sometimes be low. Therefore, in analyzing hydrogen bonds we have only considered geometric parameters involving non-H atoms (Fig. 3 ▸). The change in hydrogen-bond length, 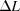, is measured as the difference in the distance from donor to acceptor between optimized and experimentally determined crystal structures. Hydrogen-bond orientations are measured using all angles involving the donor atom, acceptor atom and non-H atoms bonded to the acceptor and donor: this gives up to four angles per hydrogen bond. We measure signed changes in hydrogen-bond length and absolute changes in angles.

**Figure 4:**
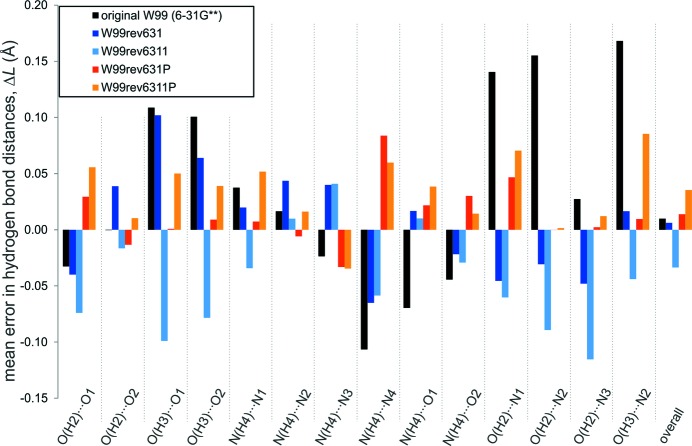
Average signed errors in hydrogen-bond lengths, 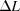, for the validation set energy minimized using each of the force fields.

The mean errors in hydrogen-bond lengths are small (< 0.04 Å) in all force fields (Table 3 ▸) and do not show an improvement in the re-parameterized force fields compared with the original W99. These mean errors vary slightly between hydrogen-bond types (Fig. 4 ▸), but show less variation in the re-parameterized models. This tighter distribution of errors is apparent in the standard deviations of the errors in hydrogen-bond lengths, which decreases with the use of the larger basis set for electrostatics and is smallest in the models using polarized electrostatics (W99rev631P and W99rev6311P).

**Figure 5:**
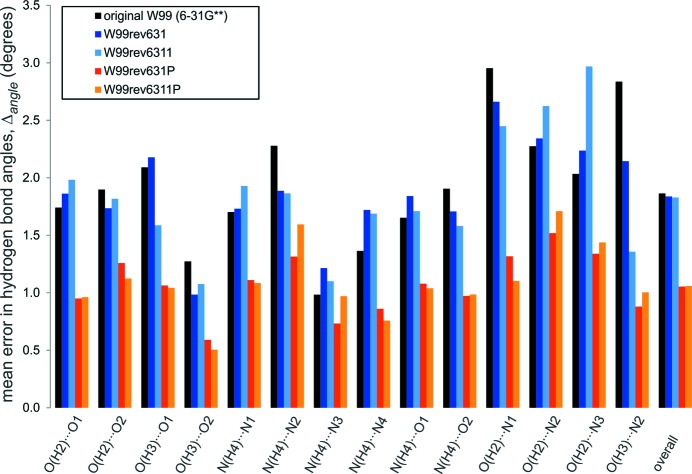
Changes in hydrogen-bond angles when energy minimized using each of the force fields, averaged over all structures in the validations set.

Mean errors in hydrogen-bond angles are just under 2° for the original W99, W99rev631 and W99rev6311. Neither the mean, nor the spread of errors is improved in either of these re-parameterized force fields (Table 4). However, we find that the use of multipoles derived from a polarized charge density (in W99rev631P and W99rev6311P) nearly halve the mean errors and reduce the standard deviation of errors substantially. This improvement in modeling the orientation of hydrogen bonds is found across all hydrogen-bond types (Fig. 5 ▸). This is a result that we did not anticipate, which demonstrates the importance of polarization in defining the directionality of hydrogen-bond interactions.

### 3.5. Lattice energies   

**Figure 6:**
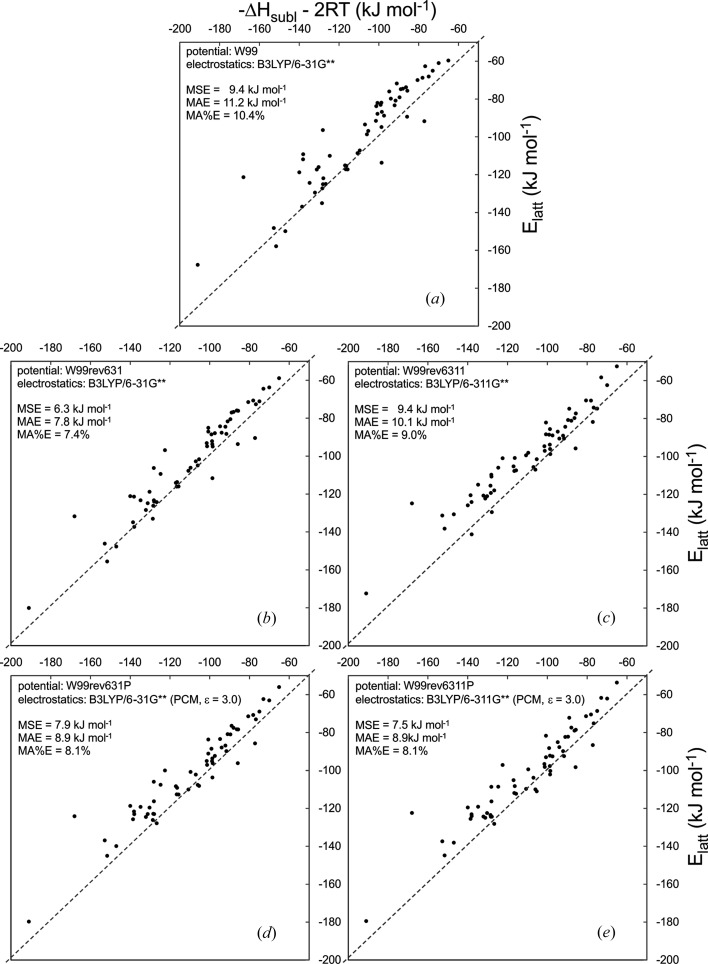
Comparison of calculated lattice energies with measured sublimation enthalpies for 59 molecular crystals from the parameterization set, using: (*a*) the original W99 force field and B3LYP/6-31G** electrostatics; (*b*) revised W99 and B3LYP/6-31G** electrostatics; (*c*) revised W99 and B3LYP/6-311G** electrostatics; (*d*) revised W99 with B3LYP/6-31G** (PCM, 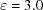) electrostatics and (*e*) revised W99 with B3LYP/6-311G** (PCM, 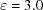) electrostatics. Mean absolute errors (MAE) and mean signed errors (MSE) are shown for each force field.

Lattice energies compare well with measured sublimation enthalpies with all of the force fields (Fig. 6 ▸). Mean absolute errors (MAE), when compared with 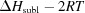, of 10.4%, or 11.2 kJ mol^−1^, with the original W99 decreased slightly in all of the re-parameterized force fields, to between 7.4 (W99rev631) and 9.0% (W99rev6311).

The thermal contribution to sublimation enthalpies [2*RT* in equation (8)] was ignored in the original parameterization of W99 (Williams, 1999 ▸, 2001*a* ▸,*b* ▸). As a result, the force field systematically underestimates the lattice energy, when compared with 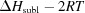, with a mean *signed* error (MSE) of 9.4 kJ mol^−1^. This systematic underestimation of lattice energy is maintained in the re-parameterized force fields (Fig. 6 ▸), with mean signed errors ranging from 6.3 kJ mol^−1^ (W99rev631) to 9.4 kJ mol^−1^ (W99rev6311). The rigid-molecule approximation used in this work contributes to these errors, since our lattice energies do not include the intramolecular strain induced by crystal packing (Thompson & Day, 2014 ▸). The nature of polarization is also likely to contribute to the systematic underestimation of lattice energies; polarization is more complex than the mean field polarization that is described by the PCM models used here, which likely miss some of the stabilizing induced interactions around strongly polar functional groups.

While we had hoped for a greater improvement in lattice energies after re-parameterization, we recognize that the training set is dominated by geometric data and the weighting applied to sublimation enthalpies in the force field training does not give this data a strong influence on the parameters. Furthermore, since we have only re-parameterized the hydrogen-bonding interactions in the current work, the parameters describing dispersion interactions between molecules, which can be a sizeable fraction of lattice energies of organic molecules, are unchanged. Re-parameterization of the entire parameter set is probably required to reduce the systematic errors in energies.

Nevertheless, we note that these errors compare favorably with errors in lattice energies with many popular dispersion-corrected solid-state density functional theory methods. Mean absolute % errors with many common DFT methods [such as PBE with TS (Tkatchenko & Scheffler, 2009 ▸) or Grimme’s (2006 ▸) dispersion correction] are reported to be in the range 10–20% (Otero-de-la-Roza & Johnson, 2012 ▸; Reilly & Tkatchenko, 2013 ▸; Carter & Rohl, 2014 ▸), although a more advanced dispersion correction, including C8 dispersion or many-body dispersion, reduces these errors to the 5–8% range.

## 4. Conclusions   

We present a revision of the W99 intermolecular force field for modeling molecular organic crystals. The force-field parameters describing hydrogen-bond interactions have been optimized to work optimally with an atomic multipole model of electrostatic interactions. We also parameterize versions of the force field that are compatible with using polarized multipoles, derived from the charge density of a molecule embedded in a continuum dielectric (PCM) approximation of the crystalline environment. Low-temperature crystal structures have been used in the re-parameterization to minimize the extent to which thermal expansion is incorporated into the empirical parameters, making the resulting force field suitable for including thermal effects, *via* lattice or molecular dynamics methods.

The re-fitting leads to important improvements in reproducing known crystal structures, as judged against a validation set of known crystal structures. Lattice parameters and densities are reproduced to within a few percent, hydrogen-bond geometries are reproduced very accurately, and we have slightly improved the agreement of calculated lattice energies with measured sublimation enthalpies. Most importantly, the re-parameterized force fields give less variation in errors between structures, modeling all types of hydrogen bonds with similar accuracies.

## Supplementary Material

Supporting figures and tables. DOI: 10.1107/S2052520616007708/gp5083sup1.pdf

## Figures and Tables

**Table 1:** Summary of the order of parameterization and the dependency of hydrogen-bond types in the training set of crystal structures

Round	Parameterized interaction	Number of parameterization structures†	Additional types present in the parameterization structures‡
1	H4⋯N1	12 (26)	
	H4⋯N2	12 (24)	
	H4⋯O1	21 (55)	
	H4⋯O2	11 (30)	
	H2⋯N1	11 (8)	
	H2⋯N2	6 (22)	
	H2⋯O1	16 (24)	
	H2⋯O2	20 (21)	
			
2	H4⋯N3	10 (10)	H4⋯O1
	H4⋯N4	12 (9)	H4⋯O1
	H2⋯N3	10 (11)	H4⋯O2, H4⋯O1, H2⋯O2
	H3⋯O2	5 (8)	H2⋯O1, H2⋯O2
			
3	H3⋯O1	24 (20)	H3⋯O2
			
4	H3⋯N1	6 (0)	H3⋯O1
	H3⋯N2	10 (31)	H3⋯O1, H3⋯O2

† The value in parentheses is the number of crystal structures in the validation set containing this hydrogen-bond combination.

‡ Parameters describing additional hydrogen-bond types present in these structures were fixed at their values from an earlier round of parameterization.

**Table 2:** Optimized values of the pre-exponential repulsion parameters, *A* (in kJ mol^−1^), for all hydrogen-bond acceptor/donor combinations in the four newly parameterized potentials The original W99 values are given for reference. These parameters are supplied in eV in the supporting information.

Acceptor atom	W99	W99rev631†	W99rev6311‡	W99rev631P§	W99rev6311P¶
	Donor atom: H2				
O1	9330	9745	12 447	12 157	14 859
O2	10 141	7429	10 131	10 999	12 640
N1	5895	12 640	14 376	15 631	18 043
N2	6079	11 964	16 017	13 315	14 473
N3	8327	11 096	15 727	12 736	13 894
N4	12 099	12 099††	12 099††	12 099††	12 099††
					
	Donor atom: H3				
O1	5278	5596	12 254	10 034	13 315
O2	5741	8587	12 833	11 192	12 447
N1	3338	9841	6754	12 833	8877
N2	3445	11 385	11 385	13 701	17 946
N3	4708	4708††	4708††	4708††	4708††
N4	6850	6850††	6850††	6850††	6850††
					
	Donor atom: H4				
O1	13 575	4631	5403	11 385	13 797
O2	14 753	11 192	10 806	14 376	13 508
N1	8587	9263	13 604	13 894	18 525
N2	8848	7719	7429	12 447	14 569
N3	12 119	4149	3280	5596	4921
N4	17 609	18 911	19 104	20 455	19 008

† The W99rev631 potential is combined with atomic multipoles derived from a B3LYP/6-31G** charge density.

‡ The W99rev6311 potential is combined with atomic multipoles derived from a B3LYP/6-311G** charge density.

§ The W99rev631P potential is combined with atomic multipoles derived from a B3LYP/6-31G** charge density calculated within a polarizable continuum model (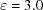).

¶ The W99rev6311P potential is combined with atomic multipoles derived from a B3LYP/6-311G** charge density calculated within a polarizable continuum model (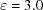).

†† Interactions that were not re-parameterized retain the original W99 repulsion parameters.

**Table 3:** Mean errors and standard deviations in hydrogen-bond lengths and angles after lattice-energy minimizations of the validation set of crystal structures using the re-parameterized force fields Changes in hydrogen bonds when using the original W99 potential (with B3LYP/6-31G** atomic multipoles) are shown for reference.

	Distances (Å)		Angles (°)	
Potential	Mean error	Std dev.	Mean error	Std dev.
W99 (6-31G**)	+0.010	0.125	1.86	2.03
W99rev631	+0.006	0.106	1.84	1.81
W99rev6311	−0.034	0.099	1.83	2.18
W99rev631P (PCM)	+0.014	0.069	1.05	1.36
W99rev6311P (PCM)	+0.037	0.069	1.06	0.85
