# Supplementary material for: An optimized intermolecular force field for hydrogen-bonded organic molecular crystals using atomic multipole electrostatics
Source: Acta Crystallogr B Struct Sci Cryst Eng Mater. 2016 Jul 16;72(Pt 4):477–87. doi: 10.1107/S2052520616007708 (PMC4971546; doi:10.1107/S2052520616007708)
Supplement: Supplementary file 1 [file b-72-00477-sup1.pdf]

Supplementary Information for:

An optimized intermolecular force field for hydrogen bonded organic molecular crystals using atomic multipole electrostatics

Edward O. Pyzer-Knapp, Hugh P. G. Thompson and Graeme M. Day

Contents:

1. Original W99 force field parameters
2. Revised parameters in eV
3. Chemical diagrams and Cambridge Structural Database reference codes for the parameterization set of crystal structures
4. Chemical diagrams and Cambridge Structural Database reference codes for the validation set of crystal structures
5. Sublimation enthalpy data used in parameterization and validation

Original W99 force field parameters

Atom type definitions are:

H1: hydrogen in G-H group<br>H2: hydrogen in alcoholic group<br>H3: hydrogen in carboxyl group<br>H4: hydrogen in N-H group<br>C4: carbon bonded to four other atoms<br>C3: carbon bonded to three other atoms<br>C2: carbon bonded to two other atoms	N1: nitrogen with triple bond<br>N2: nitrogen with no bonded hydrogen (except triple bond)<br>N3: nitrogen with one bonded hydrogen<br>N4: nitrogen with two or more bonded hydrogens<br>O1: oxygen bonded to one other atom<br>O2: oxygen bonded to two other atoms

Refer to equation (2) for functional form. All interactions between atoms of two different types are determined by the combining rules (equations 3-5). In the revised force fields W99rev631, W99rev6311, W99rev631P and W99rev6311P, all interactions between a hydrogen and the acceptor atom of a hydrogen bond are replaced by the parameters in Table 2.

In eV and Å

Atom type	A (eV)	B ($Å^{-1}$)	C (eV $Å^6$)
H1	131.42	3.56	2.885
H2	3.74	3.56	0
H3	1.20	3.56	0
H4	7.93	3.56	0
C4	1363.64	3.60	10.140
C3	2802.12	3.60	17.637
C2	1069.96	3.60	14.874
N1	998.59	3.48	14.588
N2	1060.98	3.48	14.491
N3	1989.27	3.48	24.631
N4	4201.08	3.48	58.349
O2	2949.92	3.96	13.327
O1	2498.23	3.96	13.067

In kJ/mol and Å

Atom type	A (eV)	B (Å^{-1})	C (eV Å^{6})
H1	12680	3.56	278.37
H2	361.3	3.56	0
H3	115.7	3.56	0
H4	764.9	3.56	0
C4	131571	3.60	978.36
C3	270363	3.60	1701.73
C2	103235	3.60	1435.09
N1	96349	3.48	1407.57
N2	102369	3.48	1398.15
N3	191935	3.48	2376.55
N4	405341	3.48	5629.82
O2	284623	3.96	1285.87
O1	241042	3.96	1260.73

Re-optimised parameters, in eV (parameters in kJ/mol given in Table 2 of the main text).

Acceptor atom	W99	W99rev631	W99rev6311	W99rev631P	W99rev6311P
donor atom: H2					
O1	96.7	101	129	126	154
O2	105.1	77	105	114	131
N1	61.1	131	149	162	187
N2	63	124	166	138	150
N3	86.3	115	163	132	144
N4	125.4	125.4	125.4	125.4	125.4
donor atom: H3					
O1	54.7	58	127	104	138
O2	59.5	89	133	116	129
N1	34.6	102	70	133	92
N2	35.7	118	118	142	186
N3	48.8	48.8	48.8	48.8	48.8
N4	71	71	71	71	71
donor atom: H4					
O1	140.7	48	56	118	143
O2	152.9	116	112	149	140
N1	89	96	141	144	192
N2	91.7	80	77	129	151
N3	125.6	43	34	58	51
N4	182.5	196	198	212	197

Crystal structure (CSD) reference codes and molecular diagrams of the training set

H4...N1

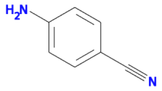

BERTOH01

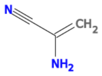

DESYOP

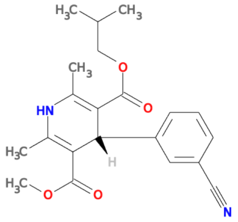

FULPIL

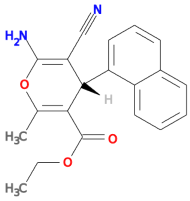

JIRSUZ

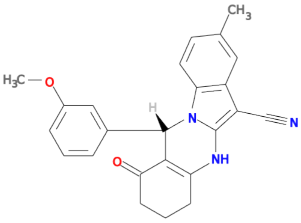

LACNAF

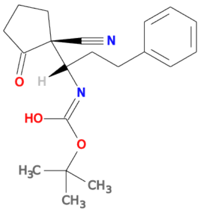

MOCQAX

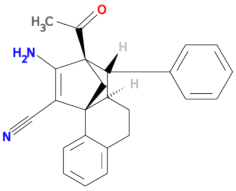

MUXXIN

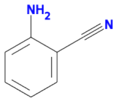

NAHQOC

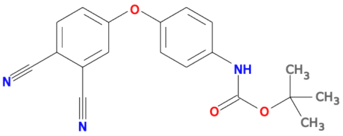

RAWCAV

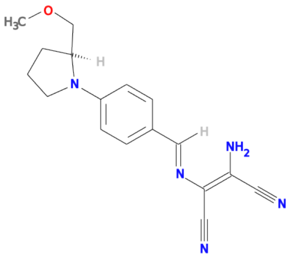

SEYSIZ

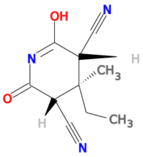

VINWOF

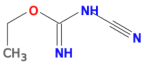

VODJEE

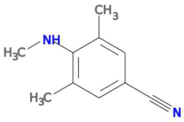

WERZUO04

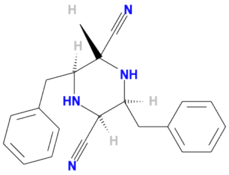

XERGAC

H4...N2

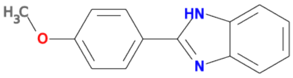

IDUGIY

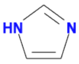

IMAZOL06

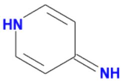

AMPYRE01

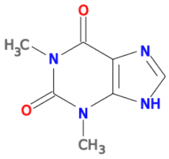

BAPLOT01

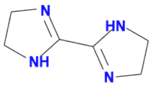

BEQWEZ

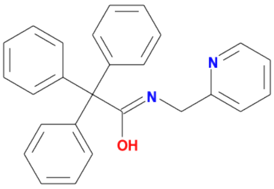

DELSUJ

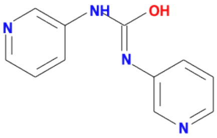

GASNEU

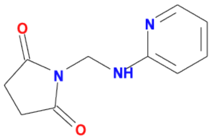

LALBAD

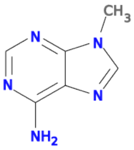

MEADEN02

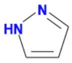

PYRZOL02

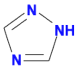

TRAZOL02

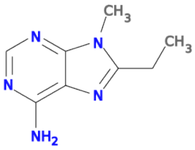

VAYJIO

H4...N3

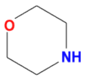

ATIZUG

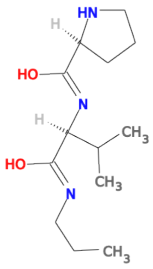

BACQON

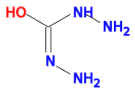

CBOHAZ02

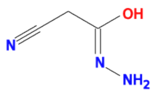

CYACHZ01

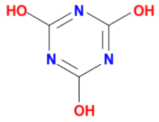

CYURAC12

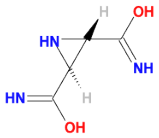

EREREY

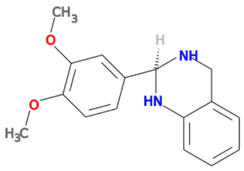

FOCYIG

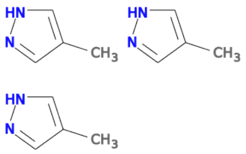

HOQHUQ

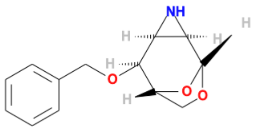

QOHBUL

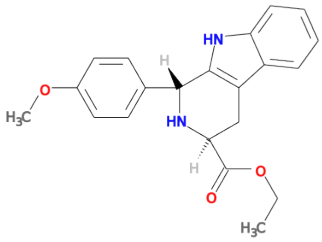

QUCPOU

H4...N4

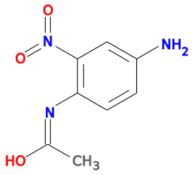

AVOJUR

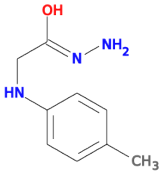

DUDGIT

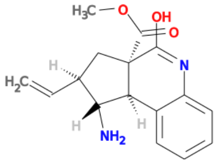

EZAZAH

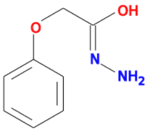

HUFDOC

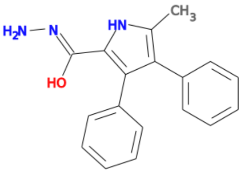

LECKUA

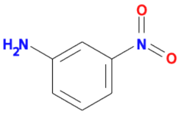

MNIANL02

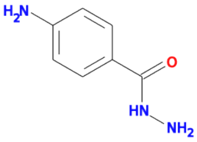

NUVXIM01

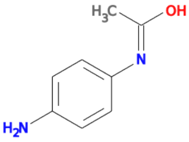

PACTAN01

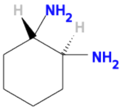

PIWXEY01

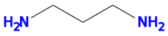

QATVUC

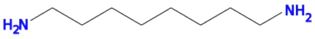

QATWUD

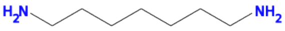

ROKZOG01

H3...N1

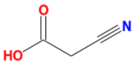

CYACAC01

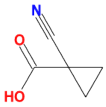

FOMWAF

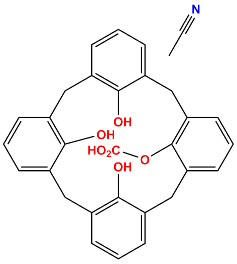

FOQMII

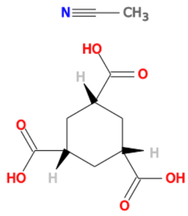

GUTRUI

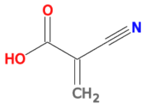

SUCSUE

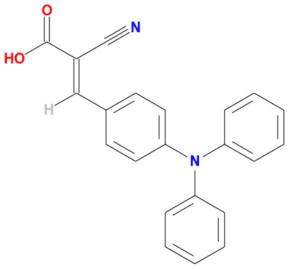

YUFRAT

H3...N2

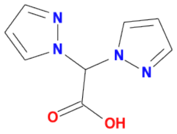

ABUBEE

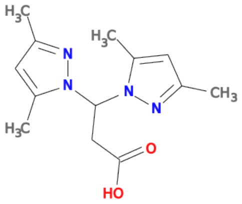

IGUWAJ

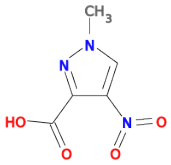

BOZGED

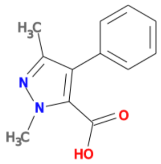

DIXMED

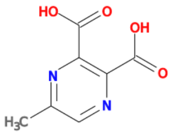

HERMOH01

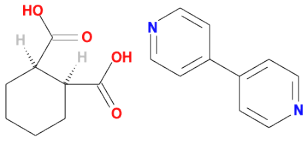

JAZBAO

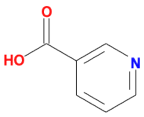

NICOAC02

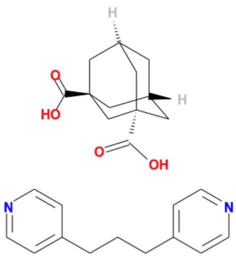

PANQOM

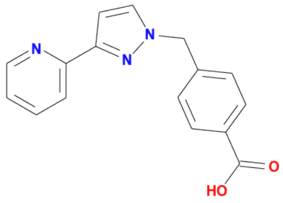

TIWBIL

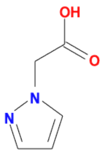

YAMFOI

H3...O1

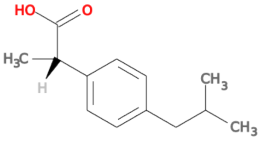

IBPRAC02

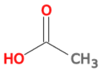

ACETAC03

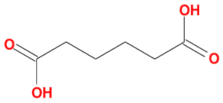

ADIPAC12

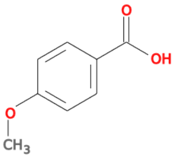

ANISIC03

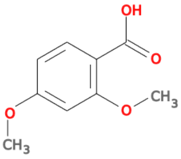

ISUQUI

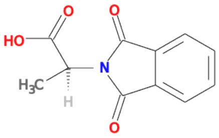

IZAMAX

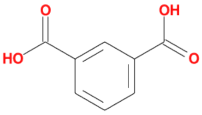

BENZDC01

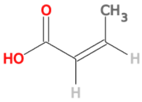

CROTAC

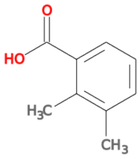

DMBZAC01

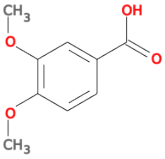

DMXBZA01

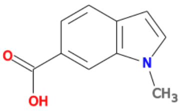

EPAGIM

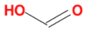

FORMAC02

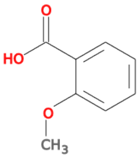

FUFBOX01

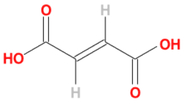

FUMAAC01

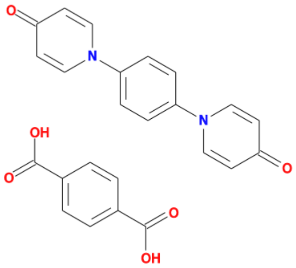

GEHTUJ

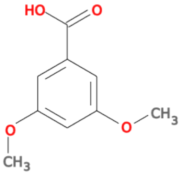

HEKMOZ

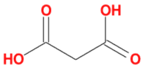

MALNAC09

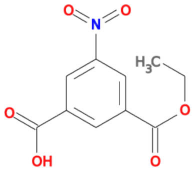

MOTCEE

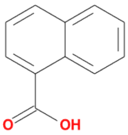

NAPOAC01

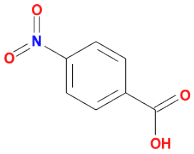

NBZOAC05

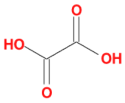

OXALAC04

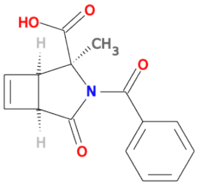

SACDIL

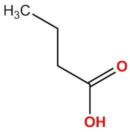

SUBRAC01

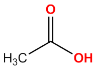

SUCACB03

H3...O2

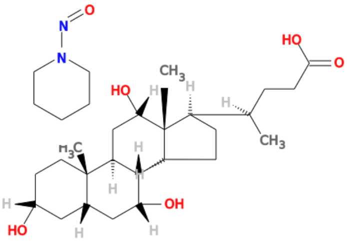

JOQHEC

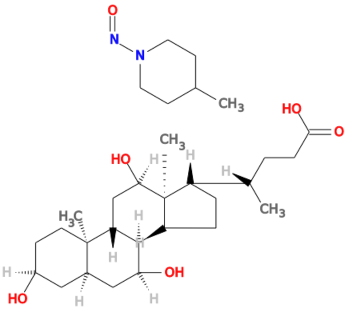

JOQHIG

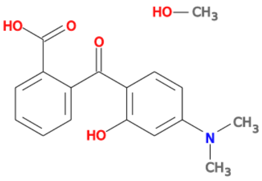

OGESEZ

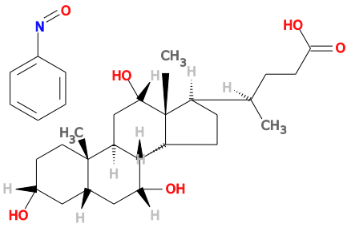

UMABAL

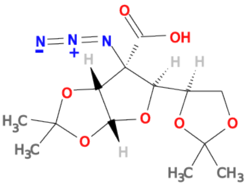

XAFMIB

H2...N1

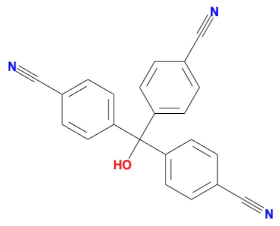

IDUDIU

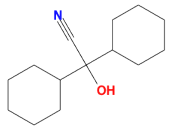

AFEVUD

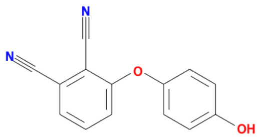

AHITOB

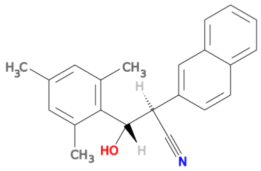

CEZNUR

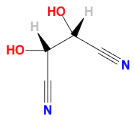

GOFQEY

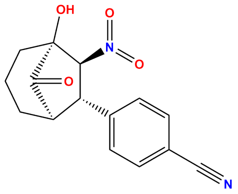

MUWRIG

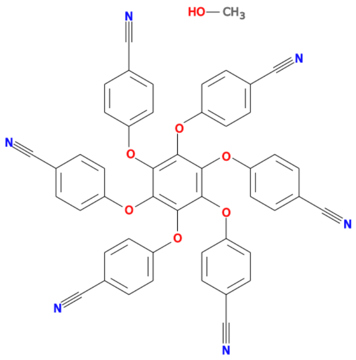

NOJZOC

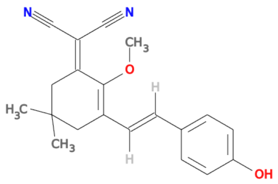

PAKBEK

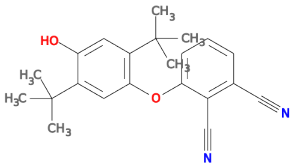

POYDOX

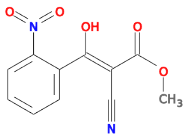

SEMYOZ

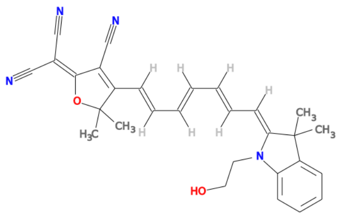

YAHGIZ

H2...N2

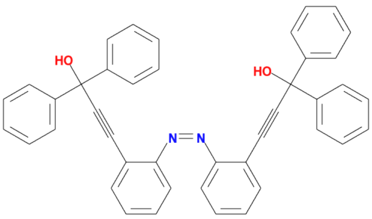

AWUGII

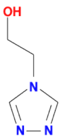

DAYMEW

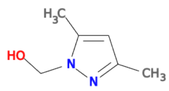

FIWFOH

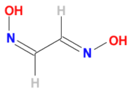

GLOXIM11

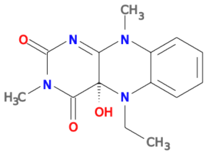

HAMSIZ

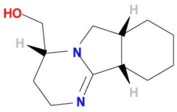

MATPEC

H2...N3

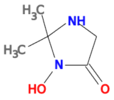

CALKOQ

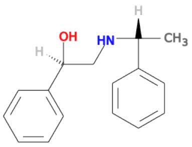

ENIYEF

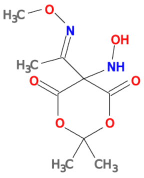

HANLEP

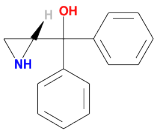

HOKCEP02

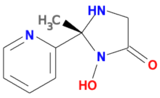

HUCZEL

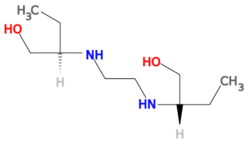

NERPOQ

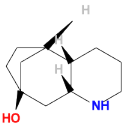

PETYOD

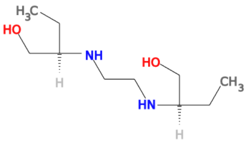

VIKMAE

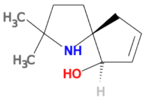

XOLPEU

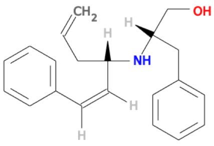

YAKXUE

H2...O1

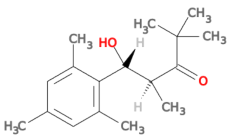

CEZPON

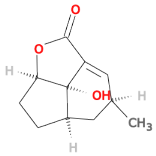

EBOVUN

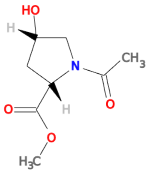

EMITEA

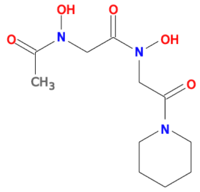

KAKYOM

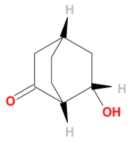

KEGHAG

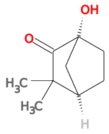

LUVCOU

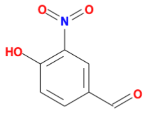

MIXXUN

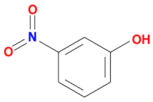

MNPHOL17

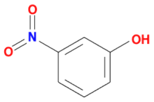

MNPHOL20

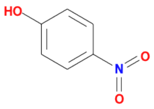

NITPOL03

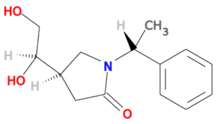

POFQEH

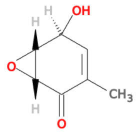

SEWHUY

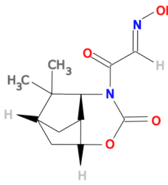

XAQTUF

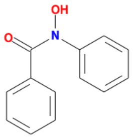

XETNOA

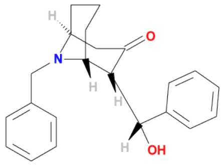

ZAPNIP

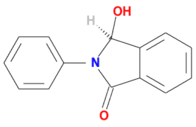

ZZZAWJ13

H2...O2

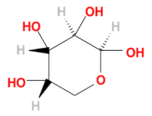

ABINOR04

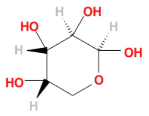

ABINOS02

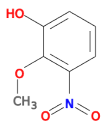

ADIPEJ

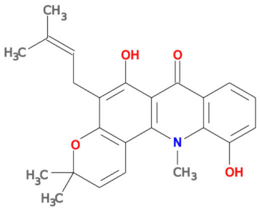

AFIHAZ

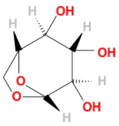

AHGLPY13

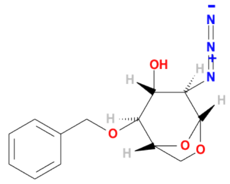

AHUZUZ

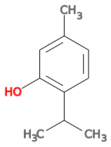

IPMEPL

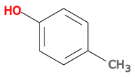

CRESOL01

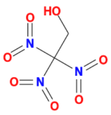

DIKXEB

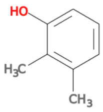

DIMPHE12

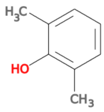

DMEPOL10

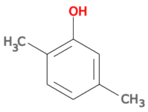

DMPHOL11

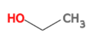

ETANOL

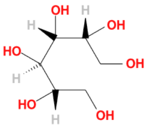

GLUCIT01

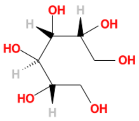

GLUCIT02

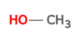

METHOL02

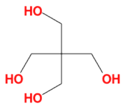

PERYTO10

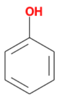

PHENOL03

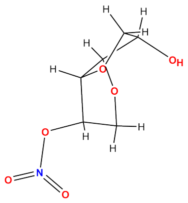

PIMKOO

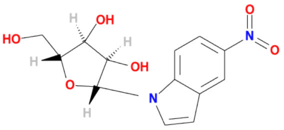

VIJFOK

Validation set structures

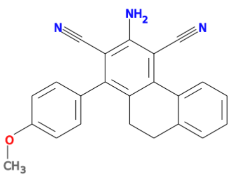

AZAJAN01

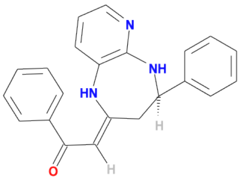

BAJYOB

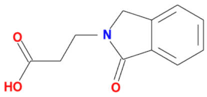

BIWWIO

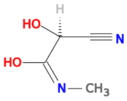

BOLZIL

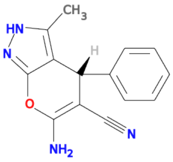

BOYZUL

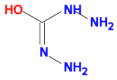

CBOHAZ01

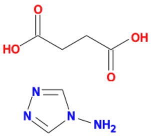

CIRXAD

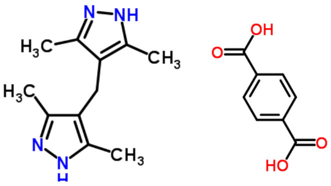

CUMRAE

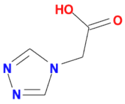

CUSDEA

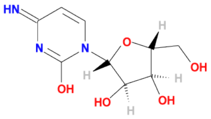

CYTIDI12

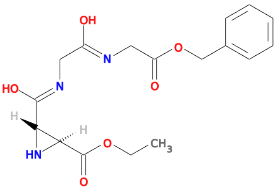

DAKCOI

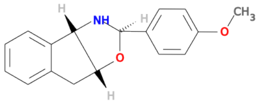

DEXSIJ

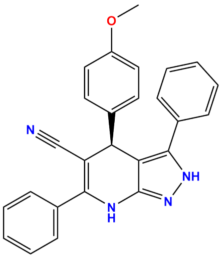

DEZQEE

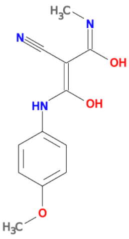

DIJGUZ

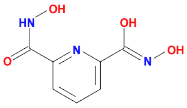

DOMQUS

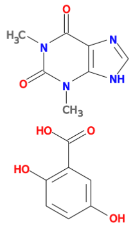

DUCROJ

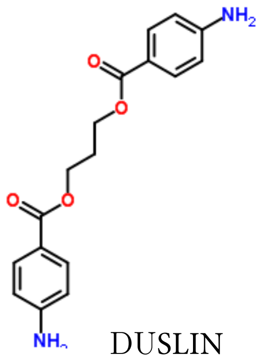

DUSLIN

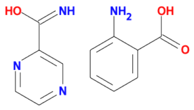

EBONUG

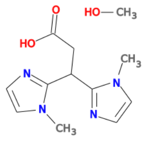

FINMEV

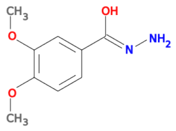

EDEVEP

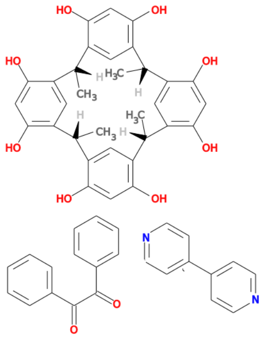

ENAZOI01

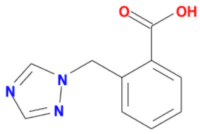

EPAGUY

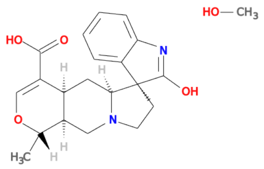

EROBUJ

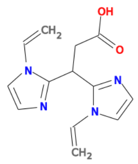

ETIXAH

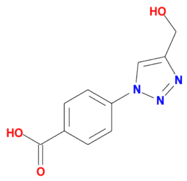

EVATUR

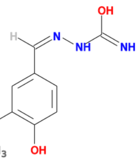

EVERED

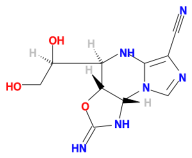

EWOSOZ

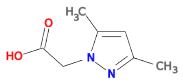

EWOWES

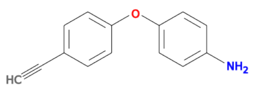

FANYAV

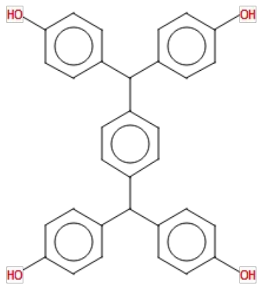

FAWXIL

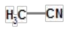

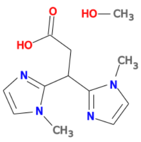

FINMEV

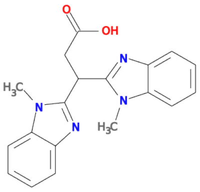

FINMIZ

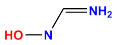

FORAMO01

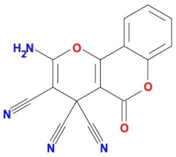

FOYBOL

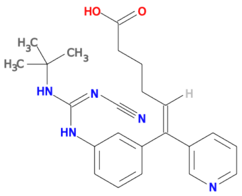

FUGQON01

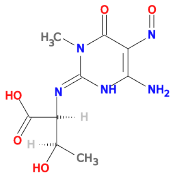

FUQLAE

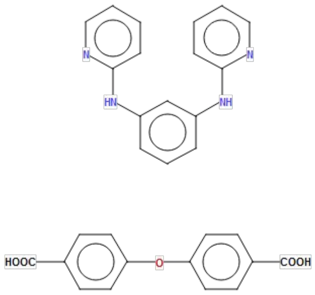

GABZAL

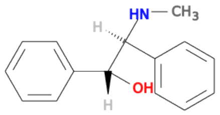

GAQXUT

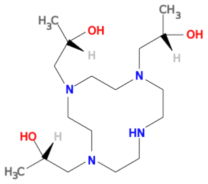

HESKUM

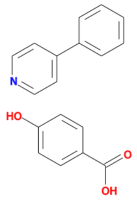

HONTAG

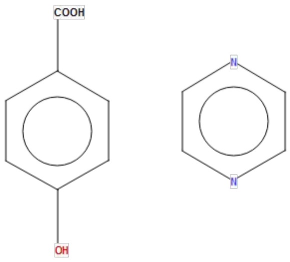

HONTEK

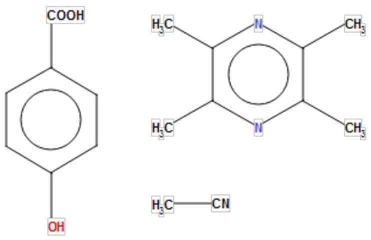

HONTIO

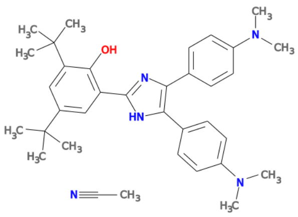

HORQOV

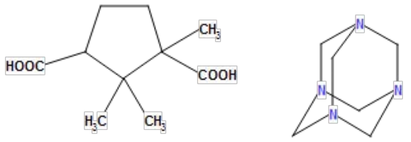

HUSWIB

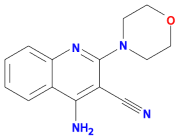

HUVBAB

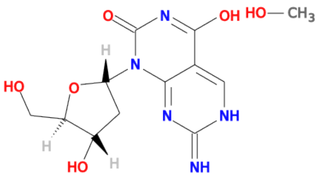

IZEYUI

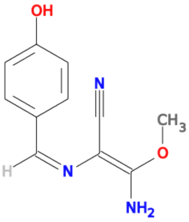

JIXBAT

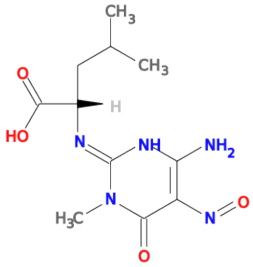

KASWUX

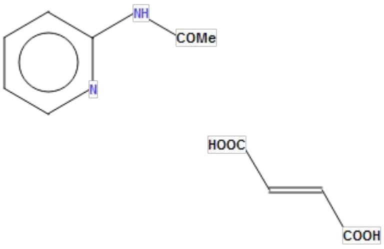

KEFBAZ

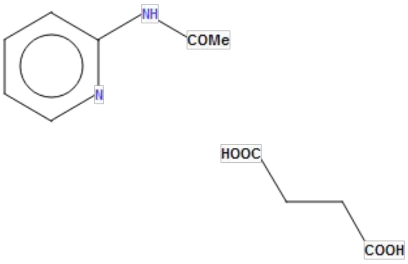

KEFBED

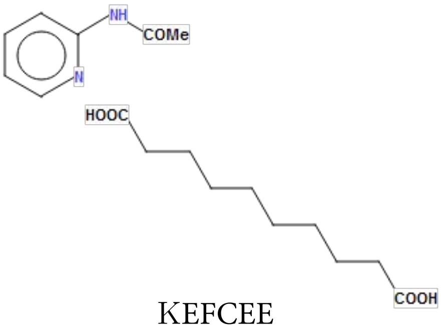

KEFCEE

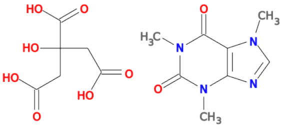

KIGKER

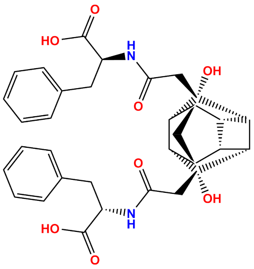

KUZJOF

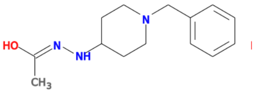

LAKYON

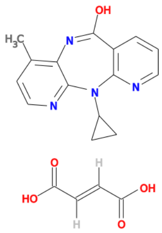

LATQII

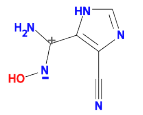

LOCTUT

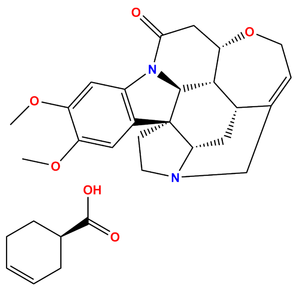

LOKRAE

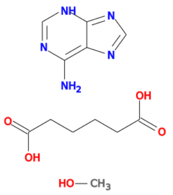

LOLCUL

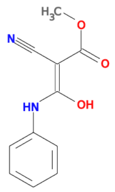

MEPTIK

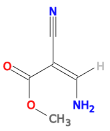

MIGWUV

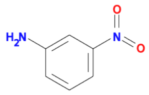

MNIANL03

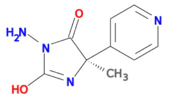

MOTFIL

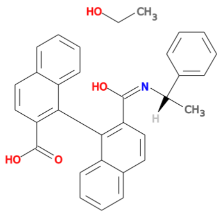

MOVRUL

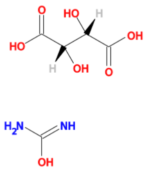

NEHPIZ01

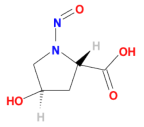

NERNII

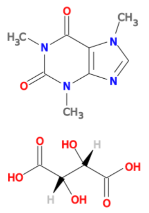

NEXWUJ

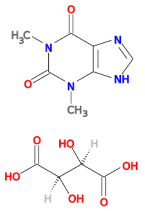

NEYCIE

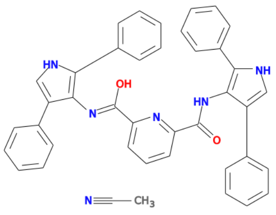

NIQDIB

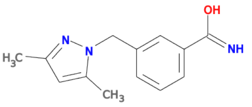

NIWWOG

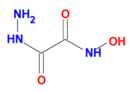

NUPTIC

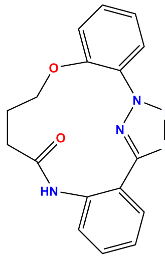

OKUZEA

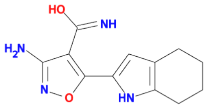

OMEKIB

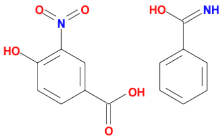

OVIBAX

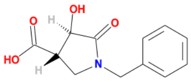

OXESIU

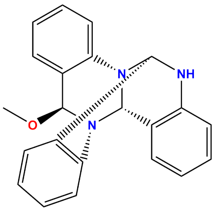

OYAKOP

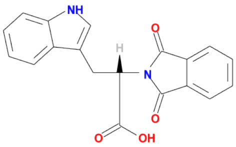

OZITAT

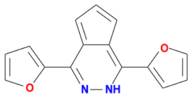

PAMBAH01

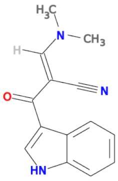

PODREG

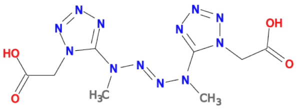

PORXUQ

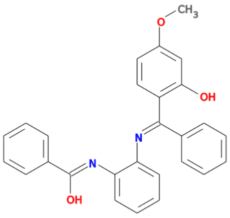

POSMIU

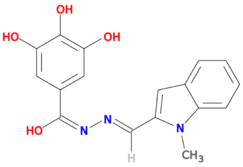

POWXEF

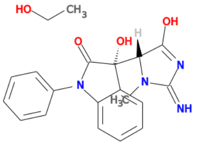

PUFGAZ

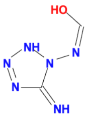

PUHJIM

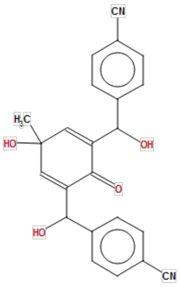

PUKZAX

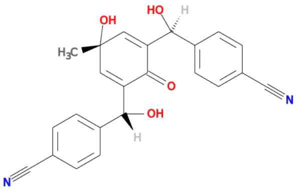

PUKZOL

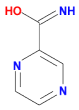

PYRZIN15

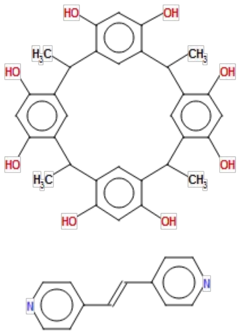

QOQDUV

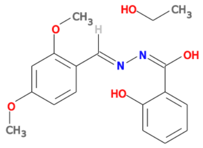

RIYRUN

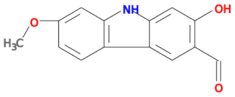

SABBII

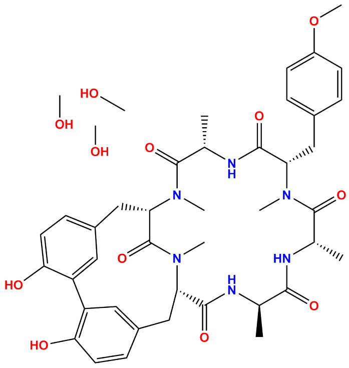

SAVLUY

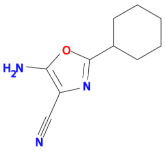

SAWPEN

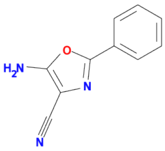

SAWPIR

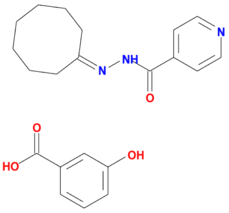

SAYNUD

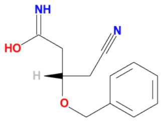

SEMWIR

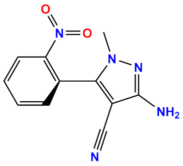

SEMZAM

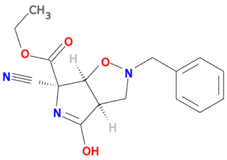

SOCBOC

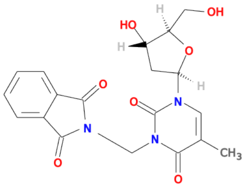

SOLLAH

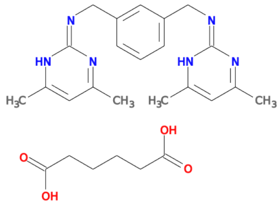

TACTAU

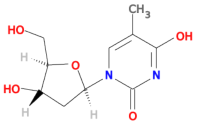

THYDIN04

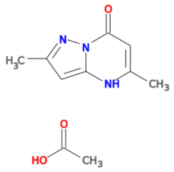

TUWYUG

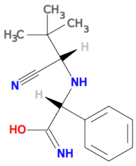

UBIMOH

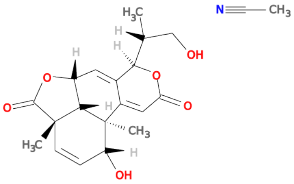

UHATAZ

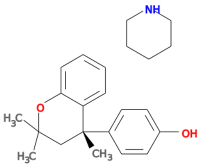

UHOXEV

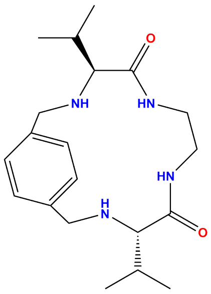

UHUSUL

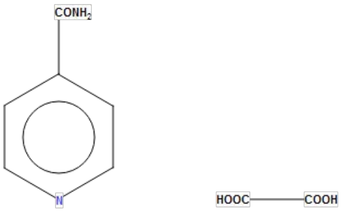

ULAWAF02

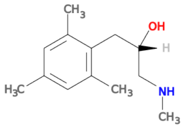

UNUBUB

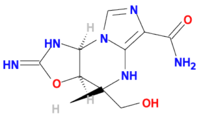

UPENOT

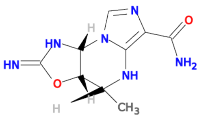

UPEPAH

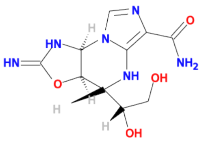

UPEPIP

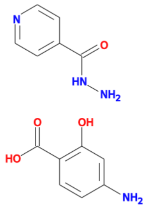

URUDER

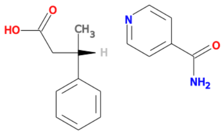

UYOSUX

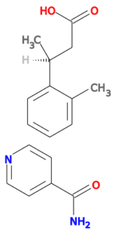

UYOTAE

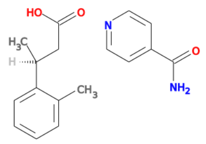

UYOTEI

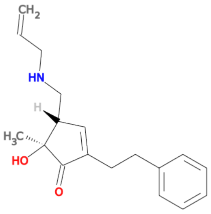

UZEVIF

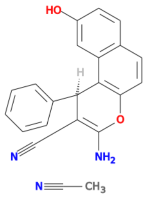

VALSOR

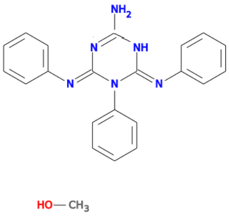

VARPUB

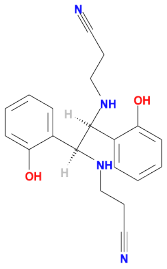

VAYXAV

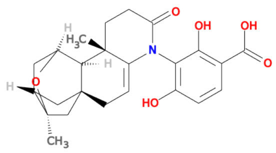

VIHTAI

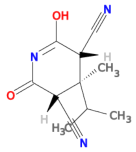

VINWUL

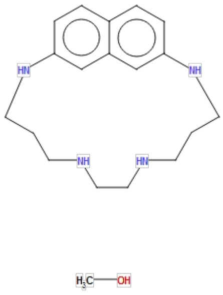

VONWEB

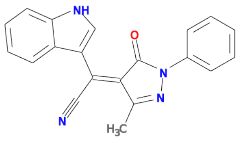

VUFJEL

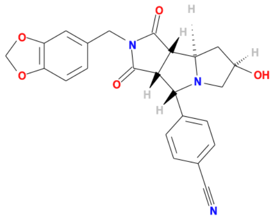

WAFBOV

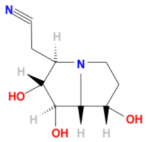

WEJVUC

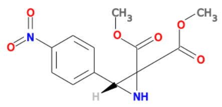

WIGJAY

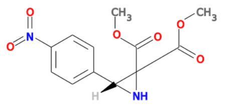

WIGJAY01

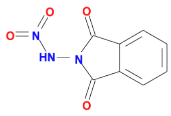

XAJSAE

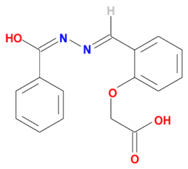

XEBWAE

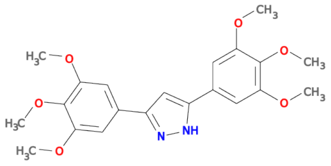

YESWUP

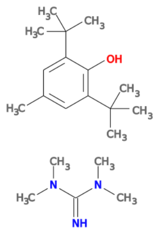

YUQYUF

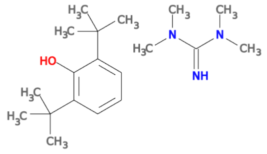

YUQYOZ

Sublimation enthalpy data used in parameterization and validation

compound	CSD reference code	ΔH_{subl} (kJ/mol)	T (K)	source
acetanilide	ACANIL02	80.6	313.5	Chickos
acetic acid	ACETAC03	67.95	298	Otero-de-la-Roza
adipic acid	ADIPAC12	133.6	298	Chickos
1,6-anhydro-β-D-Glucopyranose	AHGLPY13	125.1	365	NIST
imidazole	IMAZOL06	81.37	298	Otero-de-la-Roza
3-aminopyridine	AMIPYR	80.7	298	NIST
4-aminopyridine	AMPYRE01	87.1	298	NIST
p-methoxybenzoic acid	ANISIC03	111.6	298	NIST
thymol	IPMEPL	95.73	298	NIST
2,4-dimethoxybenzoic acid	ISUQUI	123.4	298	NIST
theophylline	BAPLOT01	135	298	NIST
isophthalic acid	BENZDC01	142	298	NIST
benzamide	BZAMID02	102	298	NIST
caprolactam	CAPLAC	89.8	298	NIST
p-cresol	CRESOL01	73.1	298	NIST
crotonic acid	CROTAC	72.2	298	NIST
cyanoacetamide	CYANAC	99.7	336.5	NIST
cyanuric acid	CYURAC12	133	298	NIST
2,3-dimethylphenol	DIMPHE12	84	298	NIST
2,2-dimethyl-propanamide	DIXTAF	89	298	NIST
2,3-dimethylbenzoic acid	DMBZAC01	104.6	298	NIST
2,6-dimethylphenol	DMEPOL10	75.6	298	NIST
2,6-dimethoxybenzoic acid	DMOXBA01	121.7	298	NIST
2,5-dimethylphenol	DMPHOL11	84.6	298	NIST
3,4-dimethoxybenzoic acid	DMXBZA01	129.8	298	NIST
formic acid	FORMAC02	60.5	274.5	NIST
formamide	FORMAM02	71.77	298	Otero-de-la-Roza
o-methoxybenzoic acid	FUFBOX01	110.7	298	NIST
fumaric acid	FUMAAC01	123.6	298	NIST
D-glucitol	GLUCIT01	186	298	NIST
glutarimide	GLUTIM	94.1	298	NIST
3,5-dimethoxybenzoic acid	HEKMOZ	127.1	298	NIST
isobutyramide	IBURAM	86	298	NIST
N-acetyl-L-	JEXNAB	133.1	298	NIST

valinamide				
maleic acid	MALIAC12	105.4	318.5	NIST
malonic acid	MALNAC09	111.4	298	NIST
9-methyl-adenine	MEADEN02	121.3	396	NIST
N-methyluracil	METURA01	121.7	385.5	NIST
methylurea	MEUREA	96.3	298	NIST Averaged value (2 data points)
m-nitroaniline	MNIANL02	96.5	298	NIST
m-nitrophenol	MNPHOL17	95.7	298	NIST Averaged value (2 data points)
4-nitroaniline	NANILI02	101	298	NIST Averaged value (8 data points)
1-naphthoic acid	NAPOAC01	112	298	NIST Averaged value (2 data points)
4-nitrobenzoic acid	NBZOAC05	119.7	298	NIST
nicotinic acid	NICOAC02	117.5	298	NIST Averaged value (3 data points)
4-nitrophenol	NITPOL03	92.4	298	NIST
oxalic acid	OXALAC04	93.63	298	NIST
oxalic acid	OXALAC06	93.7	298	NIST
pentaerythritol	PERYTO10	163	298	NIST
phenol	PHENOL03	65.3	280.5	NIST
2-pyridone	PYRIDO04	86.6	298	NIST
pyrazole	PYRZOL02	70	298	NIST Averaged value (7 data points)
suberic acid	SUBRAC01	147.8	298	NIST
succinic acid	SUCACB03	123.1	298	NIST
terephthalic acid	TEPHTH03	146.6	298	NIST
2,4,6-trinitroaniline	TNIOAN	125.3	298	NIST
1,2,4-triazole	TRAZOL02	83	298	NIST Averaged value (6 data points)
urea	UREAXX12	93.79	298	Otero-de-la-Roza
1,1-dimethylurea	WIFKEB01	93.5	298	NIST

NIST = NIST chemistry webbook, http://webbook.nist.gov/chemistry/
Chikos = J. S. Chikos and W. E. Acree, *Journal of Physical and Chemical Reference Data*, 2002, 31, 537-698
Otero-de-la-Roza = A. Otero-eRoza and E. R. Johnson, *the journal of chemical physics*, 2012, 137, 054103
